# Supplementary figures and images for: circPTPN12/miR-21–5 p/∆Np63α pathway contributes to human endometrial fibrosis
Source: eLife. 2021 Jun 16;10:e65735. doi: 10.7554/eLife.65735 (PMC8208816; doi:10.7554/eLife.65735)

# Full unedited gel for Fig. 1E

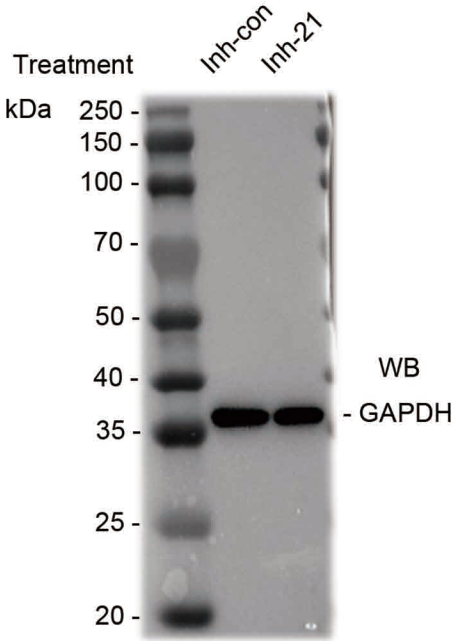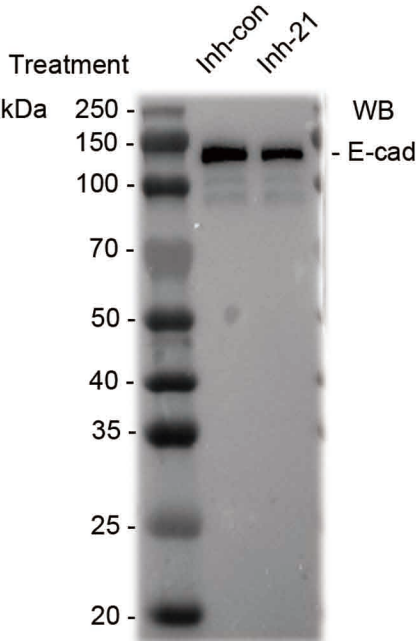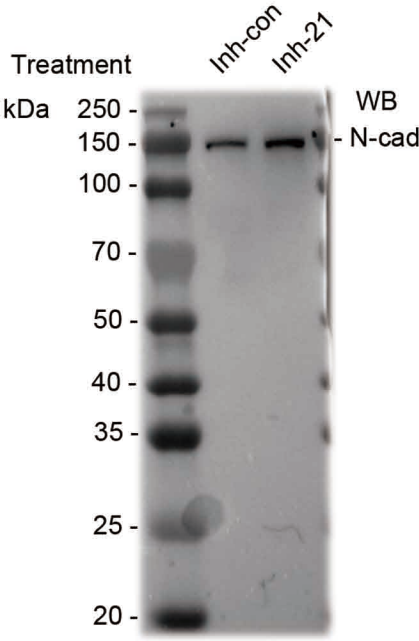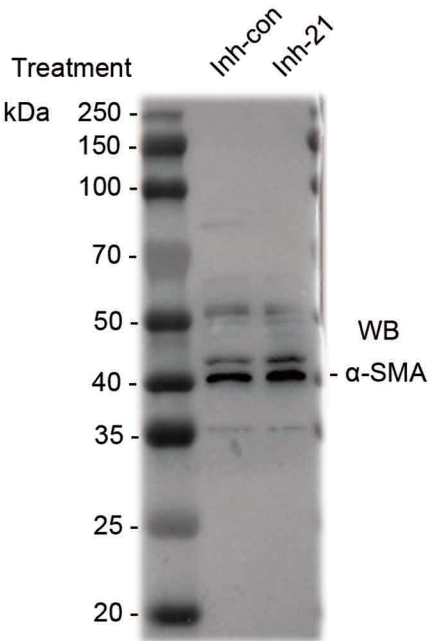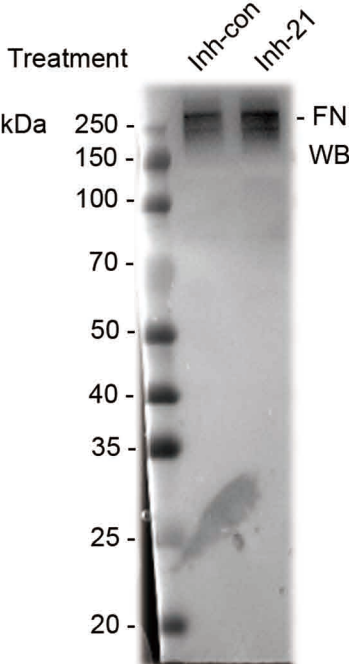

Supplement: Figure 1—source data 2. [file elife-65735-fig1-data2.pdf]

# Full unedited gel for Fig. 1F

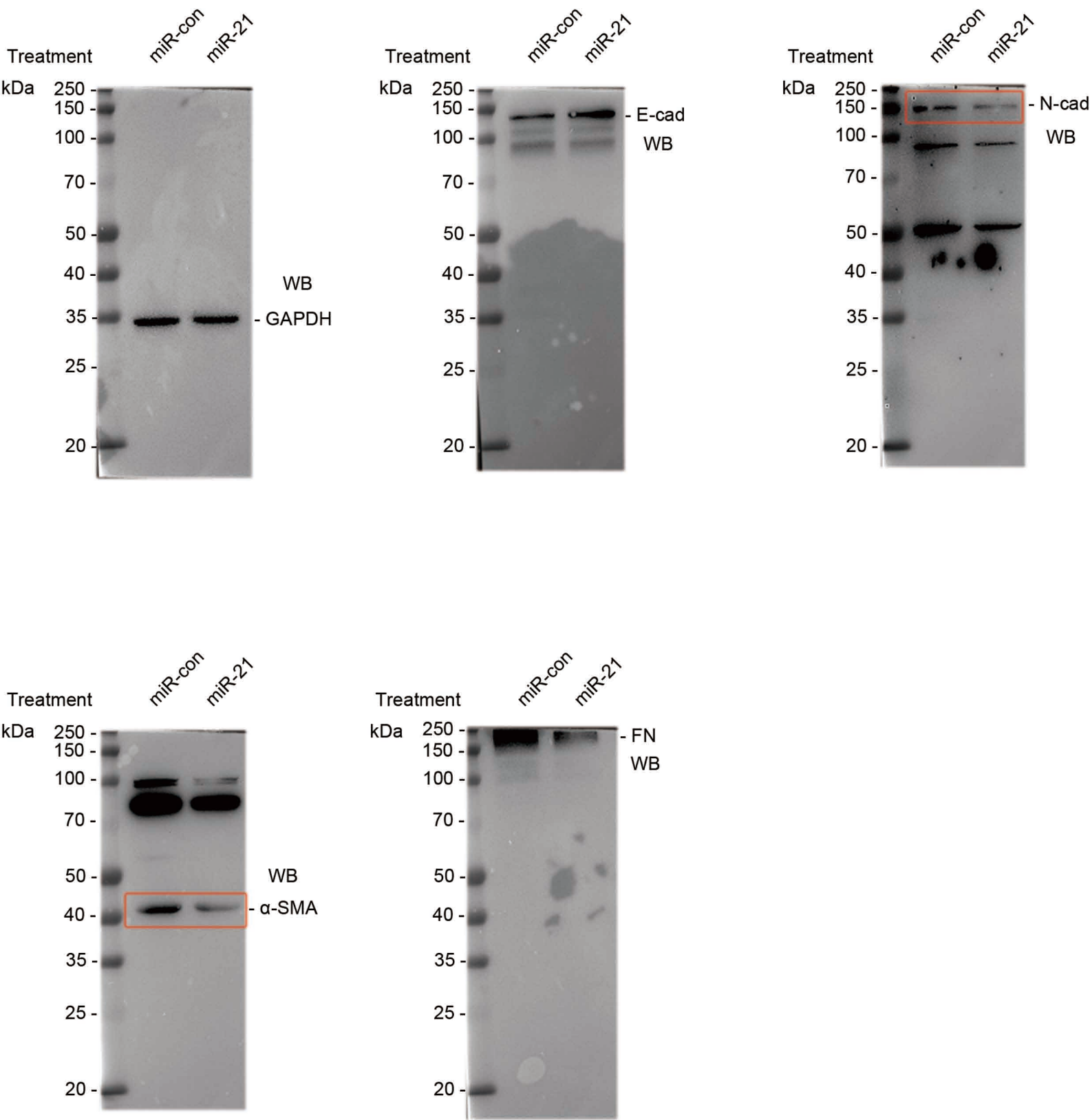

Supplement: Figure 1—source data 4. [file elife-65735-fig1-data4.pdf]

# Full unedited gel for Fig. 2F

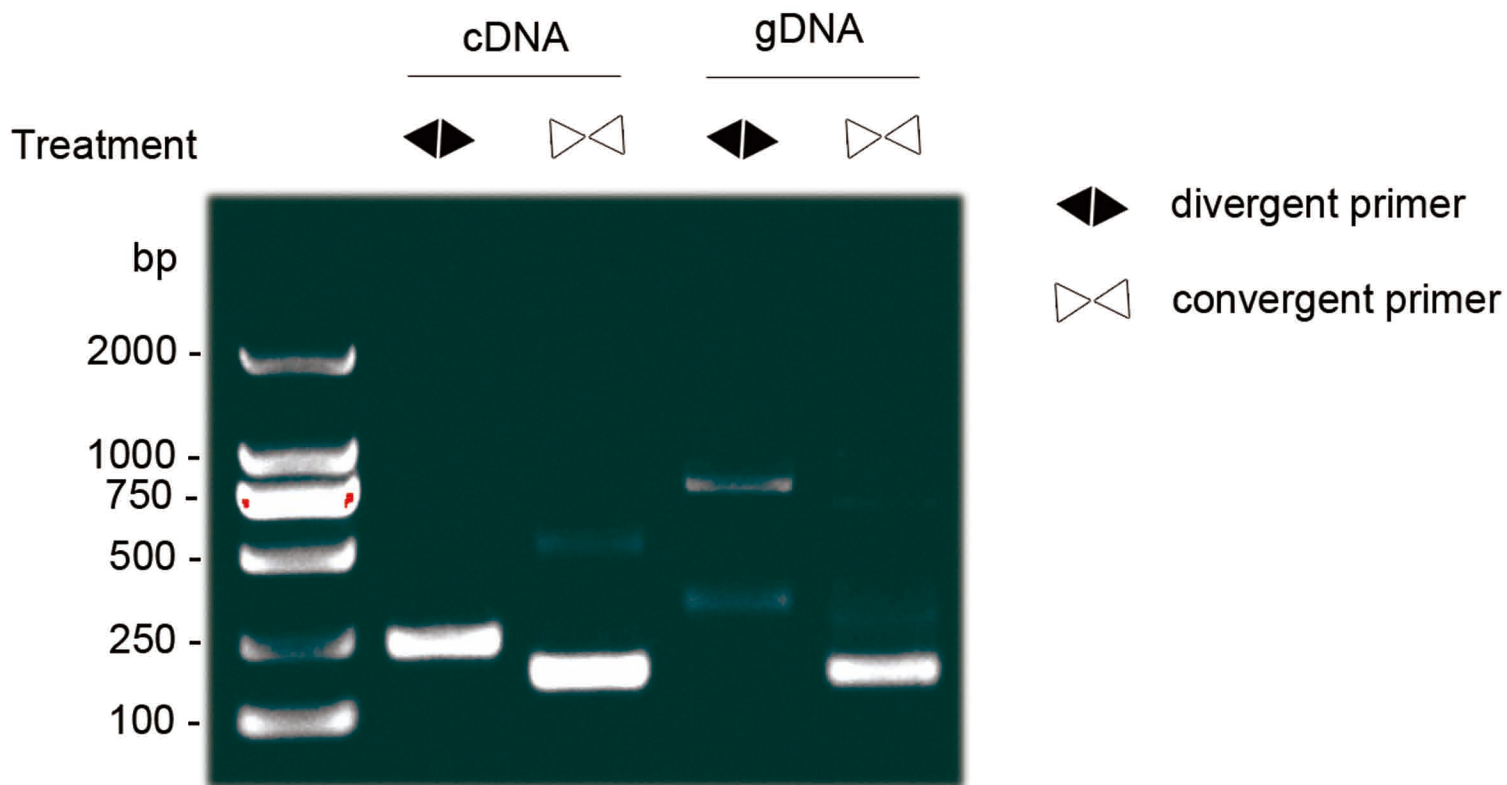

Supplement: Figure 2—source data 2. [file elife-65735-fig2-data2.pdf]

# Full unedited gel for Fig. 3I

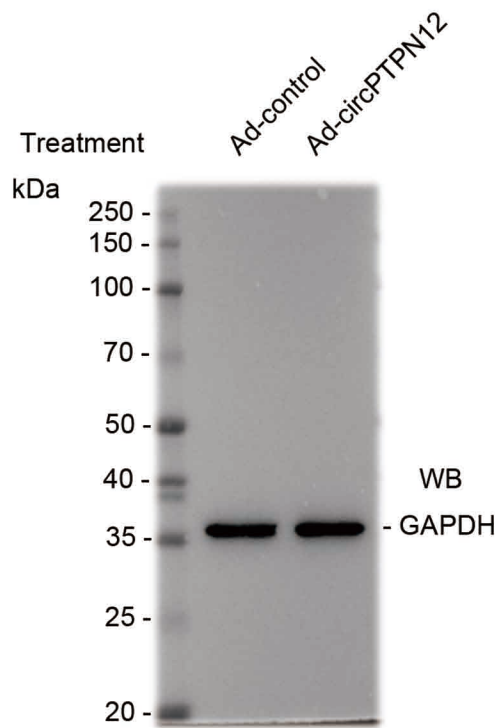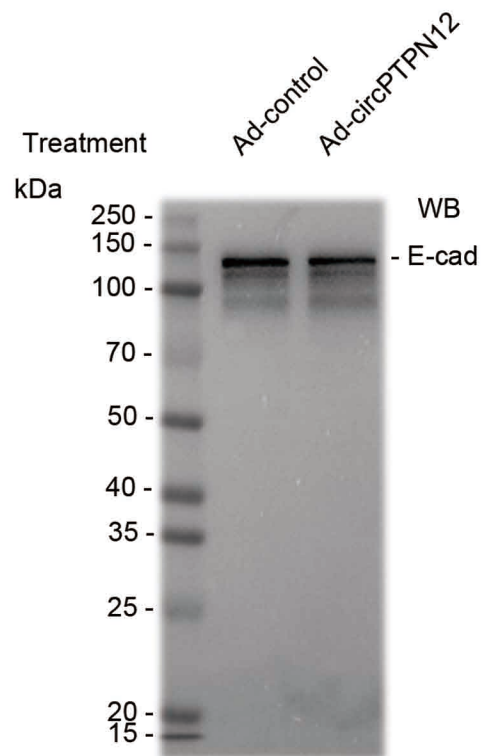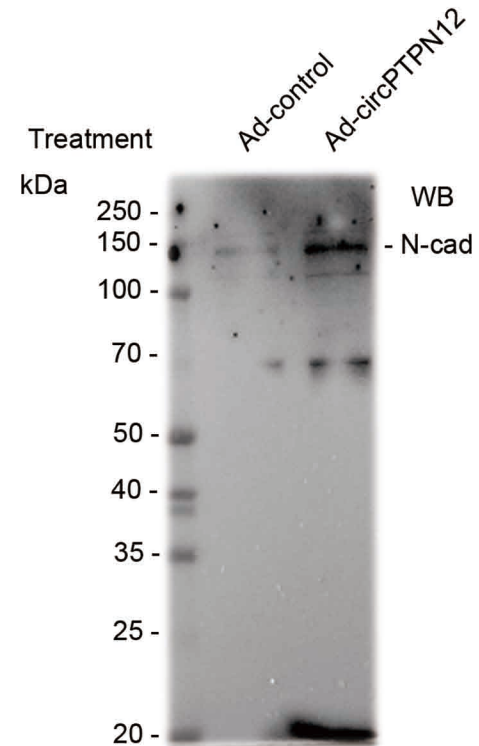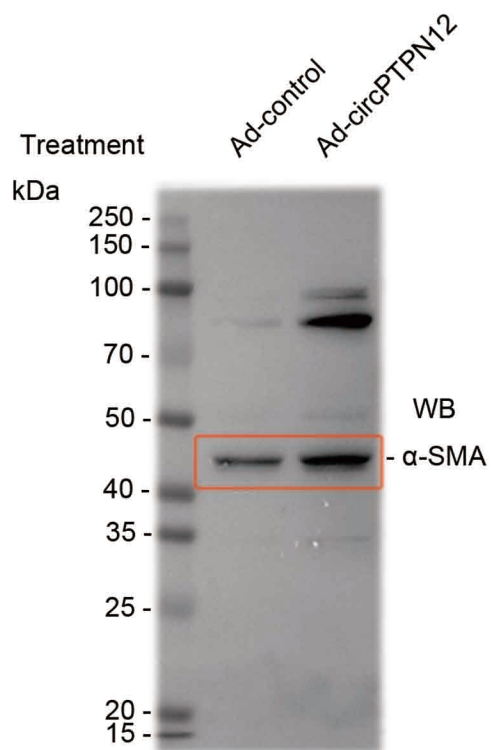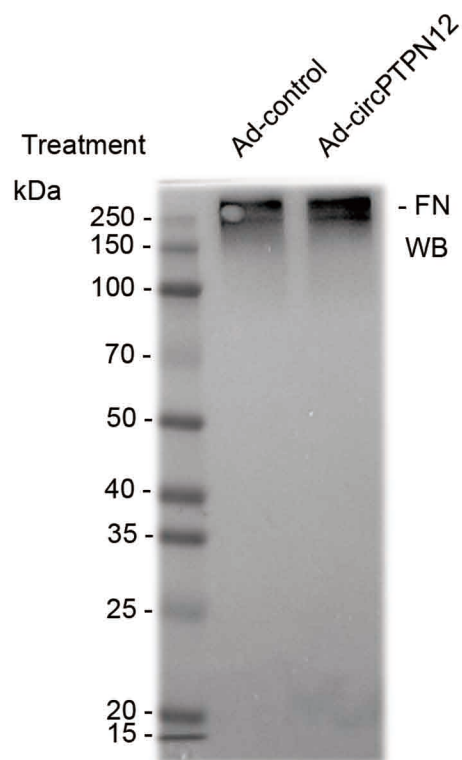

Supplement: Figure 3—source data 6. [file elife-65735-fig3-data6.pdf]

# Full unedited gel for Fig. 3J

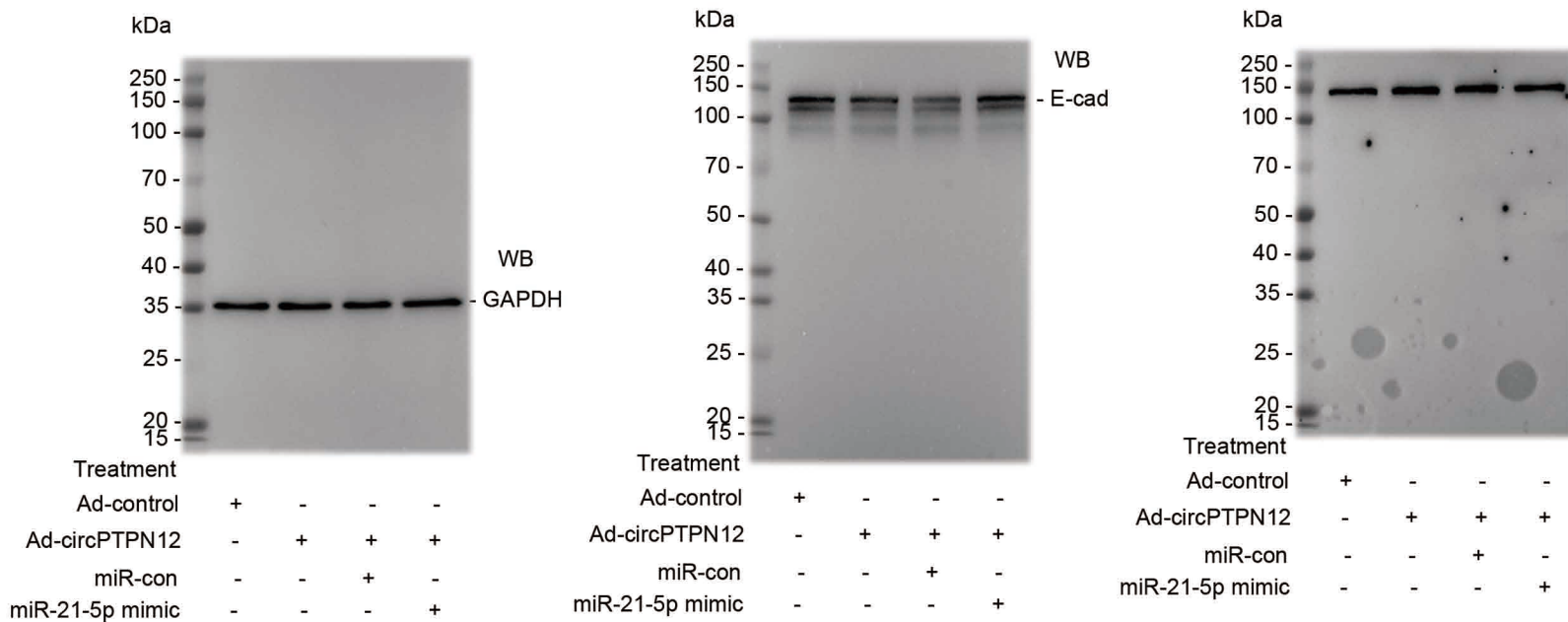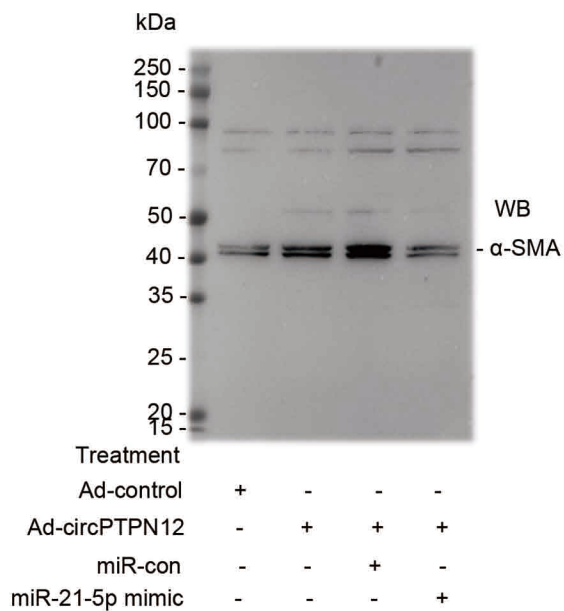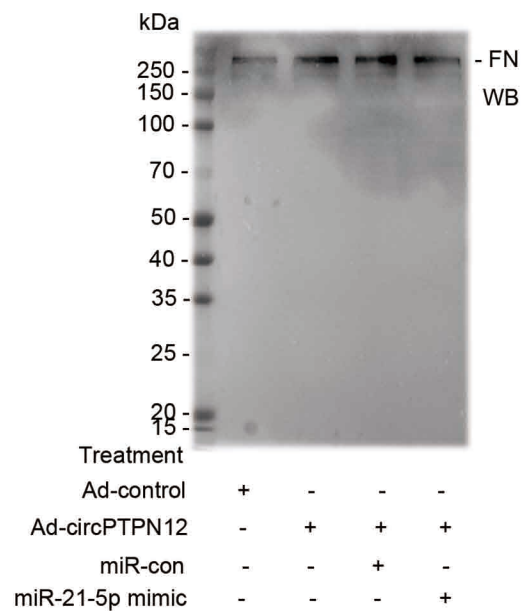

Supplement: Figure 3—source data 8. [file elife-65735-fig3-data8.pdf]

# Full unedited gel for Fig. 4, D and E

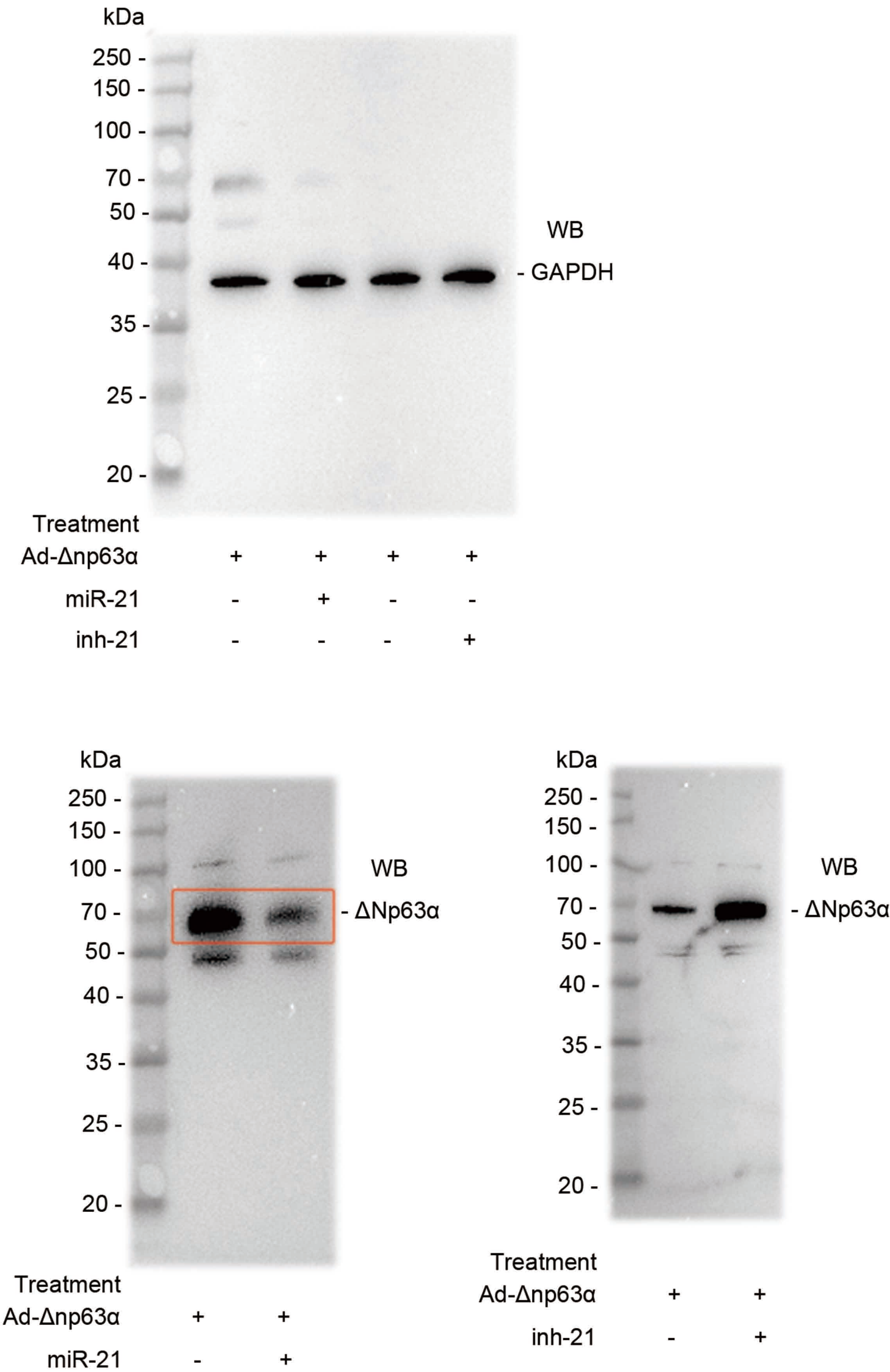

Supplement: Figure 4—source data 3. [file elife-65735-fig4-data3.pdf]

# Full unedited gel for Fig. 4G

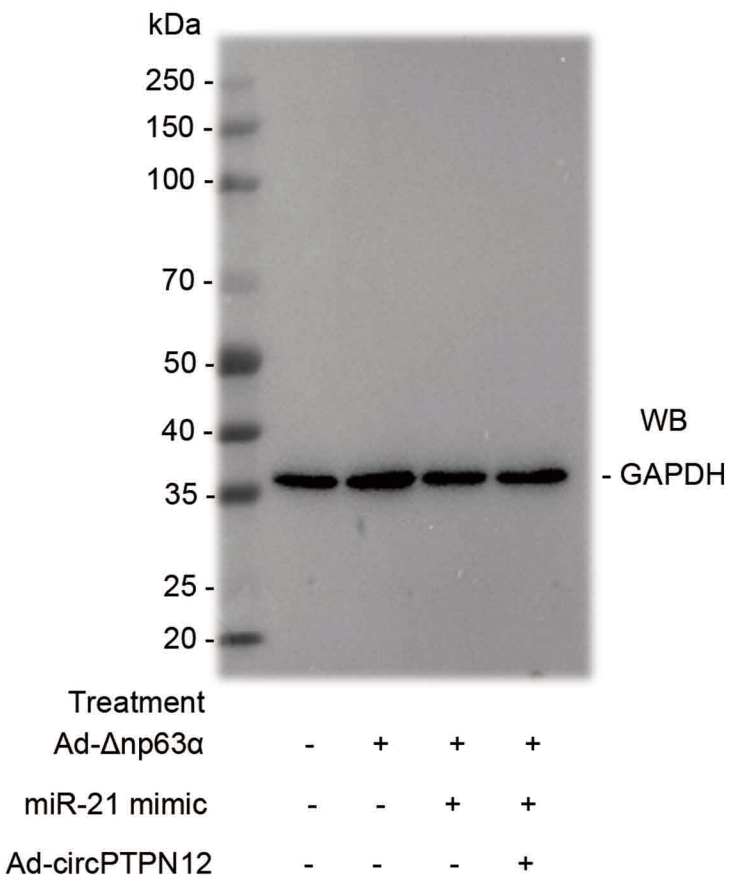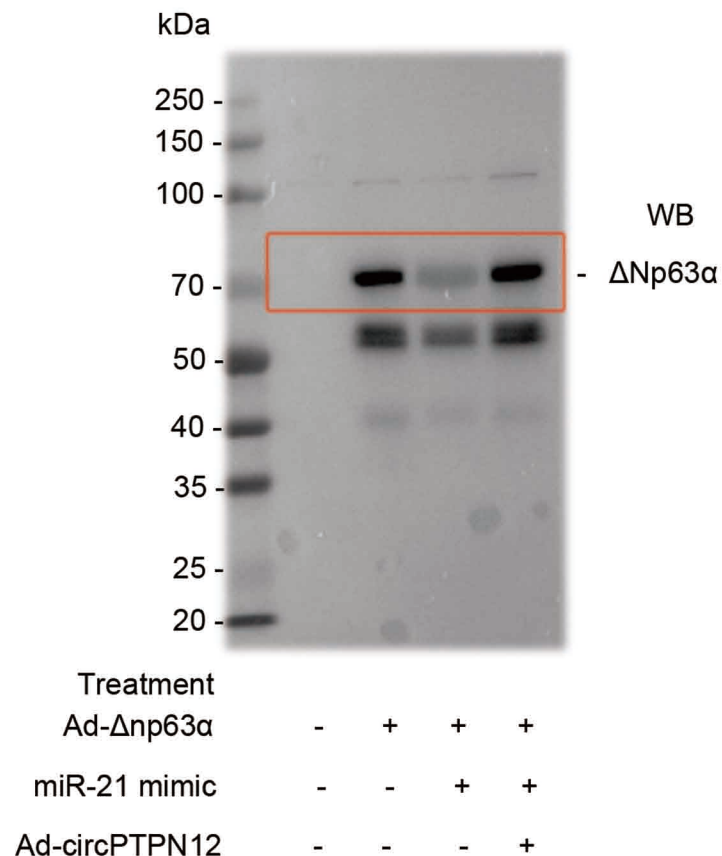

Supplement: Figure 4—source data 7. [file elife-65735-fig4-data7.pdf]

Full unedited gel for Fig. 4J

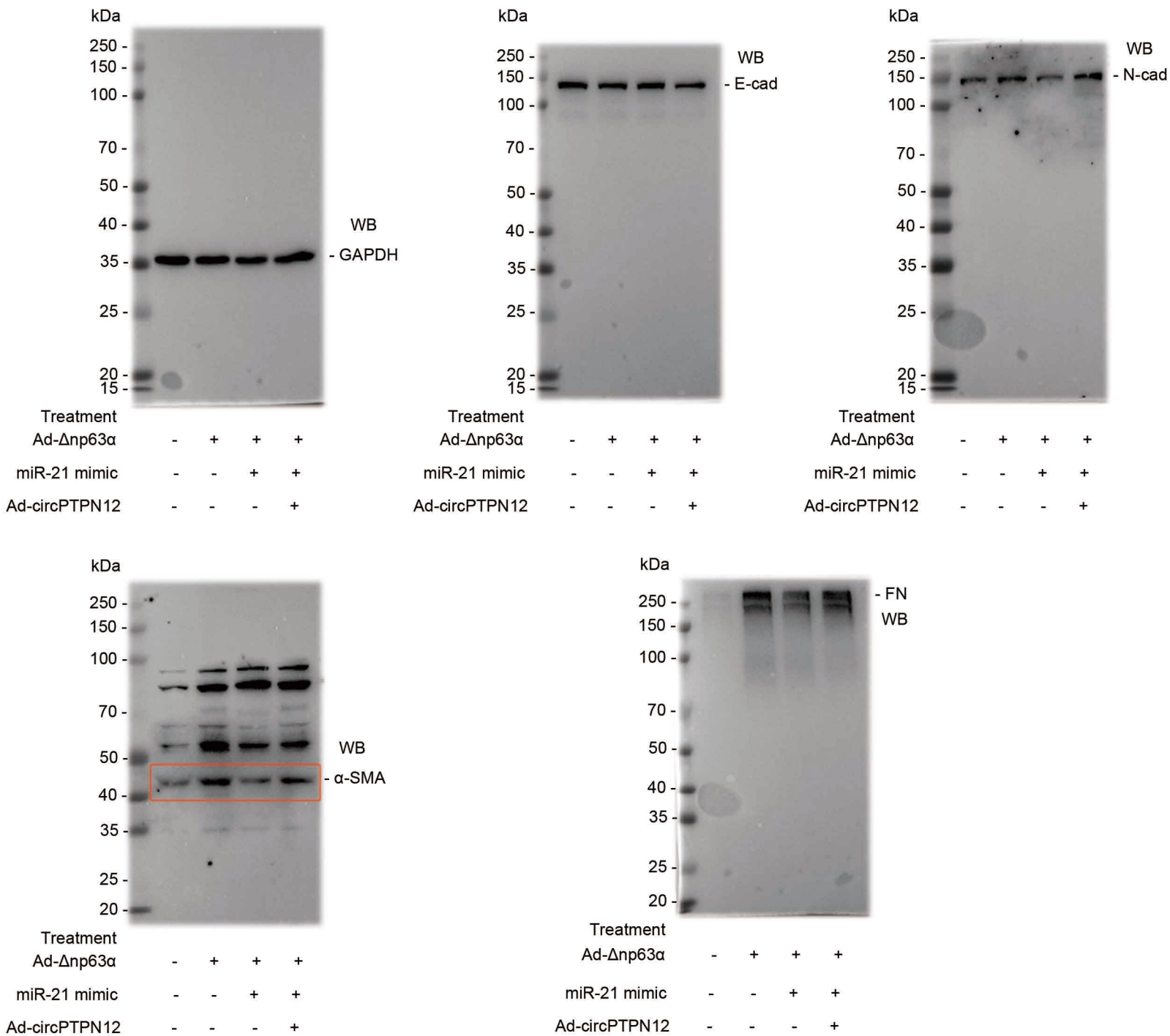

Supplement: Figure 4—source data 10. [file elife-65735-fig4-data10.pdf]
