# Supplementary material for: circPTPN12/miR-21–5 p/∆Np63α pathway contributes to human endometrial fibrosis
Source: eLife. 2021 Jun 16;10:e65735. doi: 10.7554/eLife.65735 (PMC8208816; doi:10.7554/eLife.65735)
Supplement: Supplementary file 6. [file elife-65735-supp6.docx]

Supplementary File 6. Mouse model grouping.

| Treatment | Groups |
| --- | --- |
| Mechanical injury alone | Sham-operation (*n* = 3)  Mechanically injured (*n* = 3) |
| Mechanical injury + circPTPN12 overexpression | Sham-operation (*n* = 4)  Mechanically injured + AAV-control (*n* = 4)  Mechanically injured + AAV-circPTPN12 (*n* = 4) |
| miR-21-5p replenishment | Sham-operation (*n* = 3)  Mechanically injured + AAV-circPTPN12 + agomir-NC (*n* = 3)  Mechanically injured + AAV-circPTPN12 + agomir-21-5p (*n* = 3) |
